# Advancements in Dental Care: The Evolving Landscape of Prosthetic Dentistry

**DOI:** 10.3390/jcm13051225

**Published:** 2024-02-21

**Authors:** João Paulo Mendes Tribst, Gabriel Kalil Rocha Pereira, Cornelis Johannes Kleverlaan

**Affiliations:** 1 Department of Reconstructive Oral Care, Academic Centre for Dentistry Amsterdam (ACTA), Universiteit van Amsterdam and Vrije Universiteit, 1081 LA Amsterdam, The Netherlands; 2 Faculty of Dentistry, Federal University of Santa Maria (UFSM), Santa Maria 97105-900, Rio Grande do Sul State, Brazil; gabriel.pereira@ufsm.br; 3 Department of Dental Materials, Academic Centre for Dentistry Amsterdam (ACTA), Universiteit van Amsterdam and Vrije Universiteit, 1081 LA Amsterdam, The Netherlands

## An Overview

In the dental field, the specialty of prosthodontics stands out as the frontline of innovation, continually pushing the boundaries to enhance both function and aesthetics for optimal oral rehabilitation [1]. Nothing matters if the aesthetics are good but the treatment does not last long, or if the biomaterial is reliable but the patient is not fully satisfied with the outcome. As technology advances and our understanding of oral health deepens, prosthodontists are becoming increasingly adept at bridging the gap between form and function, offering patients solutions that not only restore oral health but also improve quality of life [2]. Of course, this requires the challenging need for the prosthodontist not only to understand material science but also to be sufficiently skilled to use those materials in an optimal way [3,4].

One of the most significant clinical advancements in recent years has been the integration of digital technology into prosthodontic practice. Digital scanning, computer-aided design (CAD), and computer-aided manufacturing (CAM) have revolutionized the way dental prostheses are designed and fabricated [5]. Messy impression materials and extra time spent in pouring stone-cast models after the patient leaves are now a thing of the past. With digital workflows, we can now capture precise intraoral data, design custom restorations with incomparable accuracy, and fabricate prostheses with remarkable efficiency [6]. This not only speeds up the treatment process but also ensures optimal fit, function, and aesthetics for patients. It is a win-win situation that can only be fully achieved after a dedicated learning curve and investment in adequate digital tools [7,8].

**Figure 1:**
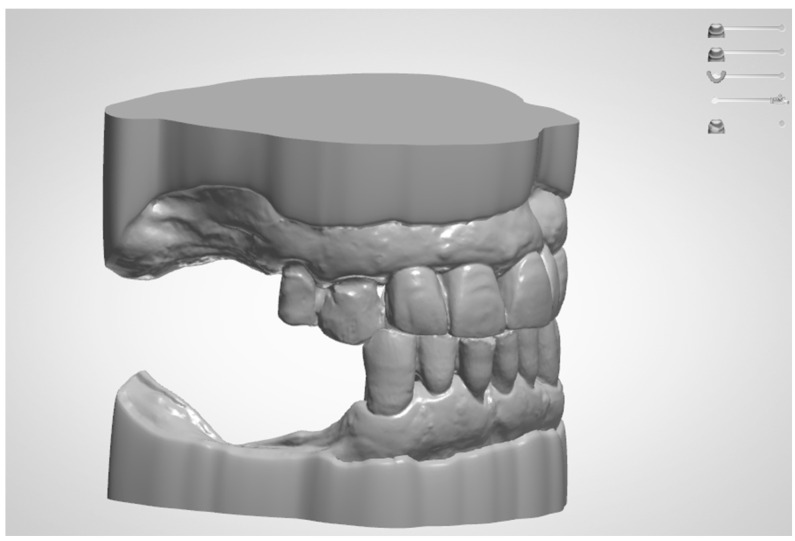
Digitalized model for a case study. With reduced steps, the impression and bite registration can be conducted in a precise workflow and the computer can be used for data storage of each virtual model.

Selecting the most adequate digital tools for your dental office is, of course, a challenge. It is not always possible to test all the machines that you want to have available to treat your patients. Careful consideration of intra-oral scanners or desktop scanners, as well as open-source software [9] and commercial licenses for CAD design, is important. Questions like how to manufacture the structure you designed—via 3-axis milling machines, 5-axis milling machines, 3D printers, or just sending it to a production center via a network—need to be addressed [10]. Will everything be integrated as a closed system, or will each component be purchased separately to create an open system? With the overflow of new information and products, it is easy to become overwhelmed and not always able to keep up with the speed of changes. What was true about digital dentistry five years ago may not be fully applicable nowadays. There are even companies developing pay-per-use CAD software that are cloud-based streaming services for the 3D design of dental structures [11]. It is not always mentioned, but the learning curve for digital dentistry starts before the first contact with digital dentistry. Understanding the lab processes from the prosthetic devices that you offer to your patients, the workflow of your dental practice, the nomenclature, and the possibilities of each apparatus are mandatory steps in avoiding headaches and reducing mistakes. Notice that avoiding all mistakes is presumptuous; it is okay to make mistakes if they do not harm anyone and do not stop your interest in digital dentistry forever (Figure 1).

**Figure 2:**
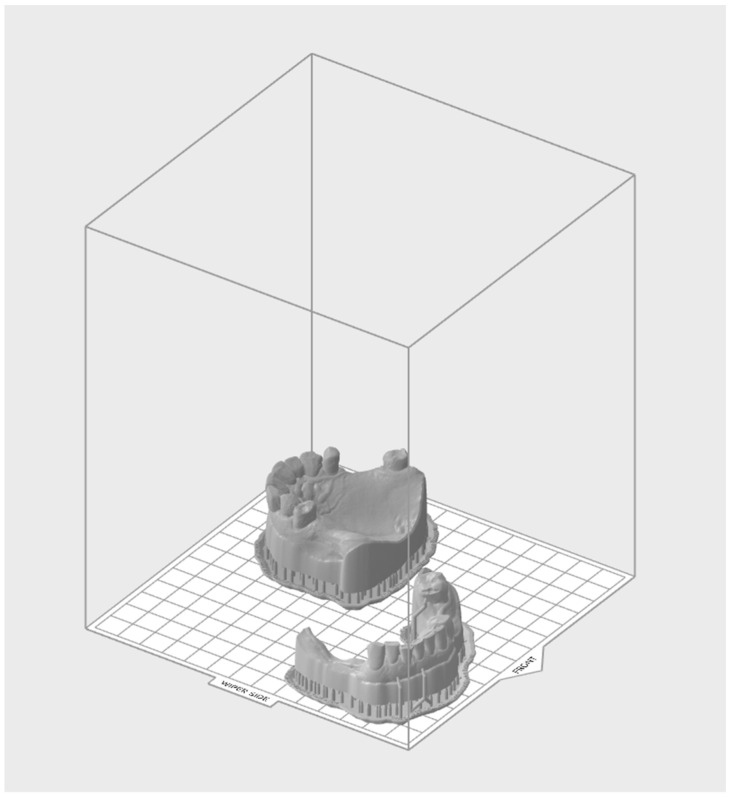
In case a physical model is needed, the 3D printing can be performed chairside, using the building platform from the 3D printer software to position the models and create the required supports.

From the previously mentioned manufacturing methods, the advent of 3D printing has further expanded the possibilities in prosthodontics [12]. This technology enables the rapid prototyping of dental prostheses, allowing for quick adjustments and modifications as needed. Whether it is crafting intricate dental crowns, bridges, or even removable dentures, 3D printing offers flexibility and customization, finally leading to better outcomes for patients, especially when speed is required. Basic dental printers can be used to manufacture surgical guides, models, mock-ups, and temporary or definitive composite restorations. With different and more robust 3D printers, the possibilities are limitless, and metal, ceramics, and biomaterials can also be manufactured in complex shapes and with optimal properties. It is also claimed to be a cleaner technology in comparison to the subtractive process, with less material consumption and waste [13]. Due to its accessibility and versatile usage, it would not be a surprise to find a 3D printer in each dental office soon (Figure 2).

In addition to technological advancements, there has been a notable shift towards a more systemic approach to prosthodontic treatment. Prosthodontists are now placing greater emphasis on considering the patient’s overall oral health instead of only aesthetic goals when planning treatment [14]. This includes conventional factors such as occlusal harmony, gingival health, and facial aesthetics, as well as reliable biomaterials, with “Green line materials” typically referring to materials or products that are environmentally friendly or sustainable. The idea is to use less hazardous materials, and more biocompatible products, and provide our patients not only with an ideal oral rehabilitation but also with the assurance that nothing will be released from the synthetic structure that is replacing the missing tissues. It is not an easy task, as we already know. Sometimes the most suitable material in terms of mechanical response is not the most adequate for fatigue, stress concentration, and/or if considering the ion leakage over time as well as the possible adverse reactions [15,16,17].

In an overview, the current research and development in prosthetic materials trend towards the introduction of advanced biomaterials that closely mimic the properties of natural tissues. It is the era of bioinspired and biomimetic materials [18,19]. Bioinspired materials are designed to mimic the structures or properties found in natural systems; they take inspiration from biological structures, such as teeth, bones, or shells, to create synthetic materials with similar characteristics [18]. For dental applications, bioinspired materials may replicate the microstructure or mechanical properties of natural teeth to improve the performance and longevity of dental restorations, such as inlays, onlays, crowns, or implants. Biomimetic materials go beyond mere imitation of natural structures and actively interact with biological systems [19]. These materials are designed to completely integrate with biological tissues or processes, promoting tissue regeneration or enhancing physiological functions. In dentistry, biomimetic materials aim to mimic the behavior and properties of natural teeth, not only in terms of structure but also in terms of response to stimuli and interaction with the surrounding oral environment. Of course, some of them may be purely merchandising for commercial strategy, but the concept is real, and the future of it is bright for patient-specific healthcare.

In conclusion, the specialty of prosthodontics is undergoing a thrilling phase of innovation and advancement. Combining the power of 3D printing and smart bioinspired/biomimetic materials, prosthodontists can shift treatment methodologies toward top-quality individualized healthcare. All of that is without mentioning the integration of artificial intelligence in the designing and manufacturing process [20]. As we enthusiastically embrace these groundbreaking developments, we edge closer to realizing the ultimate objective of restoring oral health and elevating the quality of life for patients across the globe. The question remains: are we ready to adapt to these paradigm shifts, or risk falling into obsolescence?

## References

[1] Wiens J.P., Koka S., Graser G., White S.N., Marinello C.P., Curtis D.A. (2017). Academy of Prosthodontics Centennial: The Emergence and Development of Prosthodontics as a Specialty. J. Prosthet. Dent..

[2] Ali Z., Baker S.R., Shahrbaf S., Martin N., Vettore M.V. (2019). Oral Health-Related Quality of Life after Prosthodontic Treatment for Patients with Partial Edentulism: A Systematic Review and Meta-Analysis. J. Prosthet. Dent..

[3] Munoz D.M., Kinnunen T., Chang B.M., Wright R.F. (2011). Ten-Year Survey of Program Directors: Trends, Challenges, and Mentoring in Prosthodontics. Part 1. J. Prosthodont..

[4] Chang B.M., Munoz D.M., Donoff R.B., Kinnunen T., Wright R.F. (2012). A 10-Year Survey of US Deans: Trends, Challenges, and Mentoring in Prosthodontics. Part 2: Ten-Year Survey of US Deans. Part 2. J. Prosthodont..

[5] Suganna M., Kausher H., Tarek Ahmed S., Sultan Alharbi H., Faraj Alsubaie B., Ds A., Haleem S., Meer Rownaq Ali A.B. (2022). Contemporary Evidence of CAD-CAM in Dentistry: A Systematic Review. Cureus.

[6] Mubaraki M.Q., Moaleem M.M.A., Alzahrani A.H., Shariff M., Alqahtani S.M., Porwal A., Al-Sanabani F.A., Bhandi S., Tribst J.P.M., Heboyan A. (2022). Assessment of Conventionally and Digitally Fabricated Complete Dentures: A Comprehensive Review. Materials.

[7] Hamad A. (2020). Learning Curve of Intraoral Scanning by Prosthodontic Residents. J. Prosthet. Dent..

[8] Róth I., Czigola A., Joós-Kovács G.L., Dalos M., Hermann P., Borbély J. (2020). Learning Curve of Digital Intraoral Scanning-an in Vivo Study. BMC Oral Health.

[9] Lo Giudice R., Galletti C., Tribst J.P., Melenchón L.P., Matarese M., Miniello A., Cucinotta F., Salmeri F. (2009). Vivo Analysis of Intraoral Scanner Precision Using Open-Source 3D Software. Prosthesis.

[10] Fung L., Brisebois P. (2020). Implementing Digital Dentistry into Your Esthetic Dental Practice. Dent. Clin. N. Am..

[11] Valizadeh S., Fatahi Valilai O., Houshmand M. (2022). Allocation and Scheduling of Digital Dentistry Services in a Dental Cloud Manufacturing System. Int. J. Comput. Integr. Manuf..

[12] Jeong M., Radomski K., Lopez D., Liu J.T., Lee J.D., Lee S.J. (2023). Materials and Applications of 3D Printing Technology in Dentistry: An Overview. Dent. J..

[13] Pillai S., Upadhyay A., Khayambashi P., Farooq I., Sabri H., Tarar M., Lee K.T., Harb I., Zhou S., Wang Y. (2021). Dental 3D-Printing: Transferring Art from the Laboratories to the Clinics. Polymers.

[14] Gennai S., Izzetti R., Pioli M.C., Music L., Graziani F. (2022). Impact of Rehabilitation versus Edentulism on Systemic Health and Quality of Life in Patients Affected by Periodontitis: A Systematic Review and Meta-Analysis. J. Clin. Periodontol..

[15] Munoz A., Zhao Z., Paolone G., Louca C., Vichi A. (2023). Flexural Strength of CAD/CAM Lithium-Based Silicate Glass-Ceramics: A Narrative Review. Materials.

[16] da Rosa L.S., Velho H.C., Tribst J.P.M., Valandro L.F., Kleverlaan C.J., Pereira G.K.R. (2023). Weak Adhesion between Ceramic and Resin Cement Impairs the Load-Bearing Capacity under Fatigue of Lithium Disilicate Glass-Ceramic Crowns. J. Mech. Behav. Biomed. Mater..

[17] Li X.C., Chen Z.X., Li D., Liu W.Z., Meng M. (2023). Chemical and Mechanical Stability of an Ion-Exchanged Lithium Disilicate Glass in Artificial Saliva. J. Mech. Behav. Biomed. Mater..

[18] Saratti C.M., Rocca G.T., Krejci I. (2019). The Potential of Three-Dimensional Printing Technologies to Unlock the Development of New ‘Bio-Inspired’Dental Materials: An Overview and Research Roadmap. J. Prosthodont. Res..

[19] Singer L., Fouda A., Bourauel C. (2023). Biomimetic Approaches and Materials in Restorative and Regenerative Dentistry: Review Article. BMC Oral Health.

[20] Alshadidi A.A., Alshahrani A.A., Aldosari L.I., Chaturvedi S., Saini R.S., Hassan S.A., Cicciù M., Minervini G. (2023). Investigation on the Application of Artificial Intelligence in Prosthodontics. Appl. Sci..

